# Fragmented kelp forest canopies retain their ability to alter local seawater chemistry

**DOI:** 10.1038/s41598-020-68841-2

**Published:** 2020-07-20

**Authors:** Kindall A. Murie, Paul E. Bourdeau

**Affiliations:** 1 Telonicher Marine Laboratory, Humboldt State University, Trinidad, USA; 2 Department of Biological Sciences, Humboldt State University, Arcata, USA

**Keywords:** Biogeochemistry, Biooceanography, Climate-change ecology, Conservation biology, Ecosystem services, Ecology, Biogeochemistry, Climate sciences, Ecology, Ocean sciences

## Abstract

Kelp forests support some of the most productive and diverse ecosystems on Earth, and their ability to uptake dissolved inorganic carbon (DIC) allows them to modify local seawater chemistry, creating gradients in carbon, pH, and oxygen in their vicinity. By taking up both bicarbonate and CO_2_ as a carbon source for photosynthesis, kelp forests can act as carbon sinks, reducing nearby acidity and increasing dissolved oxygen; creating conditions conducive to calcification. Recent stressors, however, have reduced kelp forest canopies globally; converting once large and persistent forests to fragmented landscapes of small kelp patches. In a two-year study, we determined whether fragmented kelp patches retained the ability to alter local seawater chemistry. We found that diel fluctuations of multiple parameters of carbonate chemistry were greater in the kelp canopy than in the kelp benthos and in adjacent urchin barrens, consistent with metabolic activity by the kelp. Further, kelp fragments increased pH and aragonite saturation and decreased *p*CO_2_ during the day to a similar degree as large, intact kelp forests. We conclude that small kelp patches could mitigate OA stress and serve as spatial and temporal refugia for canopy-dwelling organisms, though this effect is temporary and confined to daylight hours during the growing season.

## Introduction

Ocean Acidification (OA), the absorption of anthropogenically-derived CO_2_ by our world’s oceans, is well known to have negative impacts on marine organisms, particularly those that construct their skeletons and shells out of calcium carbonate (CaCO_3_)^1–3^. However, photosynthetic macrophytes (e.g., kelps and seagrasses) have the potential to modify local seawater chemistry in a way that could both buffer and intensify OA stress for calcifying organisms^4–9^. Forest-, and canopy-forming kelps are particularly good candidates for modifying local sea water chemistry because of their ability to uptake both bicarbonate and CO_2_ as a carbon source for photosynthesis, thus acting as carbon sinks, reducing nearby ocean acidity, and increasing both dissolved oxygen (DO) and providing a favorable environment for calcification by increasing the saturation states of aragonite and calcite for nearby calcifying organisms. Because kelps serve as foundational species^10,11^, supporting some of the world’s most productive ecosystems^12,13^, it is essential to understand their role as abiotic ecosystem engineers.

To date, evidence for the ability of forest-forming kelps to alter local seawater chemistry comes mainly from historically large (e.g., 10–1200 ha canopy area) and persistent kelp forests in the northeastern Pacific^14–16^, southern Pacific^6^), and Southern Ocean^17^, which are mainly dominated by giant kelp (*Macrocystis pyrifera*). However, smaller arctic *M. pyrifera* forests^18^ and shallow kelp beds dominated by the habitat-forming *Ecklonia radiata*^19^ can also drive spatial and temporal fluctuations in seawater chemistry. In southern California *M. pyrifera* forests, daily fluctuations in pH and DO have been shown to be as large as 0.36 pH units and 4.93 mg L^−1^, respectively^14^. In central California, the yearly fluctuations in pH can range from as low as 7.70 up to 8.33, coupled with fluctuations in *p*CO_2_ from 172–952 μatm^15^. Further north, large kelp forests in the Strait of Juan de Fuca, Washington comprised of both *M. pyrifera* and *Nereocystis luetkeana,* exhibit similar daily pH fluctuations with a maximum change of 0.35 pH units, with *p*CO_2_ lower by 167 μatm, and aragonite saturation higher by 0.2 units inside the kelp versus outside the kelp^16^. Because kelp forests can reduce seawater dissolved inorganic carbon (DIC) and increase pH and oxygen, they play an additional important role as a foundation species in temperate and polar coastal ecosystems, and the need to maintain these services in the future may be critical.

Threats to kelp forests have increased in frequency and severity over the past 50 years, leading to a global decline in kelp abundances of ~ 2% year^−1^^20^ and raising concern that these declines represent persistent regime shifts^21^. Analysis of kelp forest change indicate a high degree of variation in the magnitude and direction of change across the geographic range of kelps with local drivers playing an important role in driving patterns of kelp abundance^22^. Along the northern California coast, a recent ‘perfect storm’ of multiple stressors including the disappearance of a top predator (the sea star *Pycnopodia helianthoides*) and the subsequent increase in grazers (the purple sea urchin *Strongylocentrotus purpuratus*)^23^, and the recurrence of marine heat waves^24^, has caused dramatic reductions (> 90% loss in canopy cover) in historically large bull kelp (*N. luetkeana*) forests^25^. As a result, these once large and productive kelp forests have been reduced to fragmented landscapes of small kelp patches and urchin barrens. Because kelp forests provide multiple ecosystem services^26^, this large-scale deforestation has had multiple impactful cascading effects, including the mass decline (80% mortality) of abalone (*Haliotis* spp.) populations, resulting in the closure of an estimated $44M recreational fishery^27^; and the collapse of the $3M north coast commercial red sea urchin (*Mesocentrotus franciscanus*) fishery^25^. With climate change-related stressors predicted to intensify and the local extinction of purple sea urchins’ natural predator (*P. helianthoides*), continued fragmentation of northern California kelp forests seems likely.

Fragmentation of forests, whether kelp or otherwise, will lead to proportionally more edge habitat, which often experiences altered growing conditions because of novel microenvironments characterized by different temperatures and availability of resources, such as light and nutrients^28,29^. For example, in terrestrial tropical forests, interiors of intact forests retain almost 3× more carbon than edges and fragments^30^. In addition, increases in edge habitat have also been shown to be associated with 10% reductions in carbon density^31^; highlighting the importance of considering landscape fragmentation when quantifying forest effects on local carbon chemistry. In kelp forests, fragmentation alters the canopy area and the density of individuals, subsequently increasing edge habitat, which in turn alters the extent to which the forest modifies its surrounding physical environment^28^. For example, when kelp canopies are well-developed, fronds at the forest’s edge have faster elongation and larger blades, resulting in higher overall growth rates than those in the forest’s interior^28^. Carbon and nitrogen accumulation by edge fronds is also greater at this time, which leads to growth rates of edge fronds that are almost twice that of interior fronds^28^. Light availability within a kelp bed is also a function of canopy density^32^ and light in the kelp forest’s interior may be lowered to the point that nutrient uptake and photosynthesis is limited due to self-shading or lack of flow^33,34^.

With only small remnants of bull kelp forest remaining on the coast of northern California, we wanted to know whether these habitat patches maintain their ability to modify local seawater chemistry relative to nearby urchin barren habitat and to a similar extent as previously studied large, intact kelp forests in other parts of the northeast Pacific. To do so, we used a combination of in situ water samples and sensors to quantify spatiotemporal variation in seawater chemistry in the canopy and benthos of the interiors and edges of two northern California kelp forest fragments and adjacent urchin barrens.

## Results

### Kelp canopy area and habitat characterization

In 2018, the remnant kelp canopies in Trinidad Harbor (TH) and Portuguese Beach (PB) were approximately 0.32 and 1.05 ha, respectively. In 2019, we observed a reduction in kelp canopy at PB to 0.85 ha. Bull kelp density in 2018 was 3.78 and 3.28 stipes m^−2^ at TH and PB, respectively. In 2019, despite the overall reduction in canopy cover, PB’s bull kelp density increased to 4.44 stipes m^−2^. The kelp fragment in TH disappeared in the winter between 2018 and 2019, and did not return until August 2019, almost a full month after we started our sampling. Stipe density for both bull kelp and understory flora (e.g., *Pterygophora californica,* and *Laminaria *spp.) was zero in adjacent urchin barrens.

Average daily chl *a* concentration did not differ between habitats (*t* = 0.157, df = 12, *P* = 8.78). Clod card dissolution rates did not differ between habitats (ANOVA; *F*_4,9_ = 1.65, *P* = 0.246) or with the interaction between habitat x deployment depth (ANOVA; *F*_4,9_ = 1.89, *P* = 0.196), but dissolution rates did differ with depth (ANOVA, *F*_4,9_ = 36.01, *P* < 0.001). Clod cards deployed 1 m below the surface (m.b.s) lost more mass than those in the middle of the water column (Tukey HSD, *P* = 0.012), which lost more mass than those 1 m above the benthos (m.a.b.) (Tukey HSD, *P* = 0.003). Light intensity during daylight hours was above 350 (lum ft^−2^) 25% of the time in the interior of the kelp canopy, 65% of the time at the edge of the kelp canopy, and 80% of the time at the urchin barren surface. In contrast, benthic habitats reached this threshold less than 1% of the time in the kelp interior, 30% in the kelp edge, and 64% in the urchin barren.

### Within-habitat spatial variation in seawater chemistry

**Figure 1:**
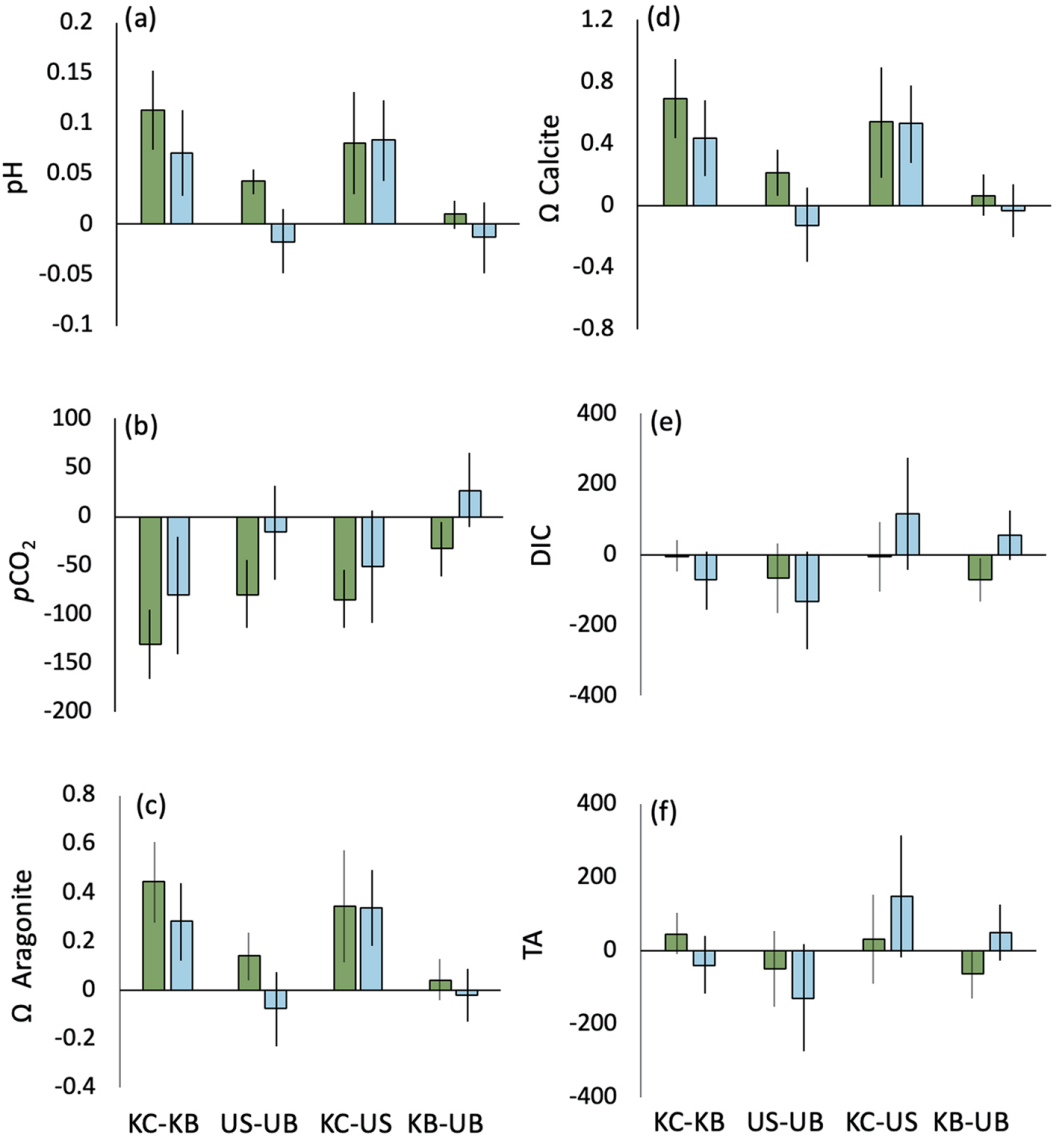
Average paired (per day) differences between habitats for (**a**) pH, (**b**) *p*CO_2_, (**c**) Ω aragonite, (**d**) Ω calcite, (**e**) DIC, and (**f**) TA, for Trinidad Harbor (blue) and Portuguese Beach (green). Error bars represent 95% C.I. KEY: *KC* kelp canopy, *KB* kelp benthos, *US* urchin barren surface, *UB* urchin barren benthos.

Seawater carbonate chemistry varied within kelp and urchin barren habitats in a manner consistent with a kelp canopy effect. On average, pH was 0.11 and 0.07 pH units higher in the kelp canopy than the kelp benthos at PB and TH, respectively (Fig. 1a). Further, *p*CO_2_ was 130.87 μatm lower in the kelp canopy than in the kelp benthos at PB and 80.85 μatm at TH, resulting in a 23 and 12% difference in *p*CO_2_, respectively (Fig. 1b). Aragonite saturation states were larger by 0.44 (PB) and 0.28 (TH) units in the kelp canopy than in the kelp benthos, and similarly, calcite saturation was 0.69 (PB) and 0.44 (TH) units higher in the kelp canopy than in the kelp benthos (Fig. 1c,d). In contrast, we saw no difference in both dissolved inorganic carbon (DIC) and total alkalinity (TA) at either PB or TH when comparing the kelp canopy and benthos (Fig. 1e,f).

Differences in seawater carbonate chemistry between the urchin barren surface and urchin barren benthos varied between sites. In the PB urchin barren, carbonate chemistry differences between the surface and benthos were in a similar direction to those in the kelp forest, but the differences were roughly half the magnitude; with pH 0.05 units higher in the urchin barren surface than the urchin barren benthos at PB (Fig. 1a). Further, *p*CO_2_ at PB was 79.92 μatm lower at the urchin barren surface than in the urchin barren benthos, resulting in a 12% difference in *p*CO_2_ (Fig. 1b). Aragonite and calcite saturation states were larger by 0.26 and 0.31 units in the urchin barren surface than in the urchin barren benthos (Fig. 1c,d). Similar to the comparison in the kelp, we saw no difference in both DIC and TA at PB when comparing the kelp canopy and benthos (Fig. 1e,f). In the TH urchin barren, carbonate chemistry was similar between the surface and the benthos (Fig. 1a–f).

### Among-habitat spatial variation in seawater chemistry

To reinforce that the paired differences in carbonate chemistry were due to photosynthetic activity by the kelp, we also wanted to compare paired differences across habitats. We found that carbonate chemistry differed inside and outside of kelp fragments in a manner consistent with a kelp canopy effect. pH was 0.08 units higher in the kelp canopy than the urchin barren surface at both PB and TH (Fig. 1a). In addition, *p*CO_2_ at PB was 84.53 μatm lower in the kelp canopy than in the urchin barren surface and 51.49 μatm lower at TH, resulting in a 20 and 11% difference in *p*CO_2_, respectively (Fig. 1b). Note that the difference in *p*CO_2_ at TH was only marginally significant. Aragonite and calcite saturation states were larger by 0.35 and 0.54 units in the kelp canopy than at the urchin barren surface at both sites (Fig. 1c,d). Similar to the comparison in the kelp and urchin barren, we saw no difference in both DIC and TA at PB and TH when comparing the kelp canopy and urchin barren surface (Fig. 1e,f).

Finally, when comparing the kelp benthos with the urchin barren benthos we found that only *p*CO_2_ and DIC were lower in the benthos at PB, while the rest of the carbonate chemistry parameters were not different. *p*CO_2_ and DIC were lower in the benthos by 7 and 4%, respectively (Fig. 1b,e).

### Temporal variation in seawater chemistry inside and outside of kelp fragments

Spatial variation was consistent with a kelp canopy effect on local seawater chemistry, but to confirm that photosynthetic activity by the kelp was driving these spatial patterns, we looked at the temporal fluctuations in pH and dissolved oxygen (DO) within all four habitats (kelp canopy, kelp benthos, urchin barren surface, urchin barren benthos).

**Figure 2:**
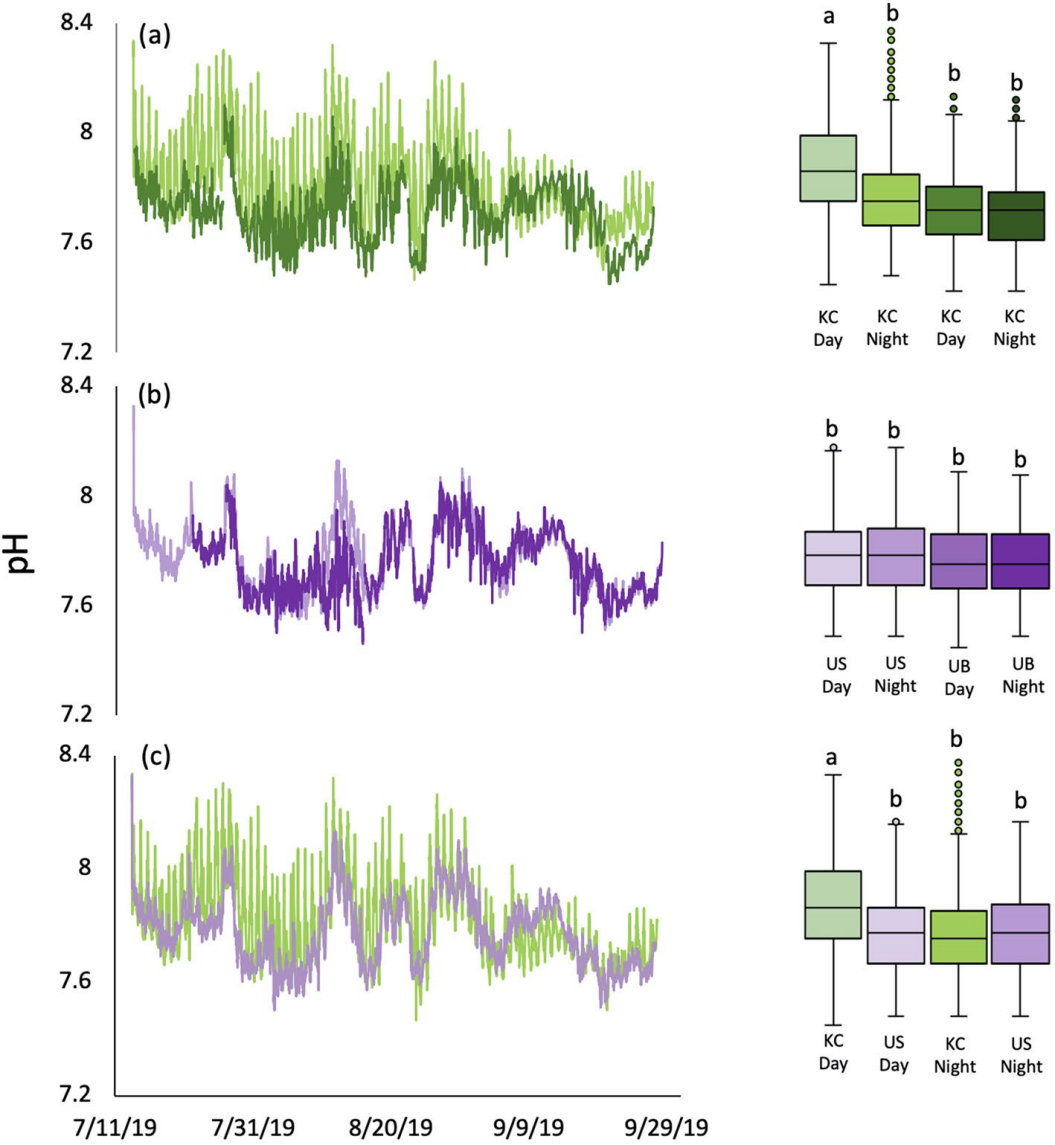
Spatiotemporal variation in pH in (**a**) kelp canopy (KC-light green) vs. kelp benthos (KB-dark green); (**b**) urchin barren surface (US-light purple) vs. urchin barren benthos (UB-dark purple); (**c**) kelp canopy (KC-light green) vs. urchin barren surface (US- light purple). Each panel includes fluctuations in pH through time (left), and average habitat-specific day and night pH’s (right). Letters denote significant pairwise differences (*P* < 0.001).

On average, pH (calculated from *p*CO_2_ and DIC values) was 0.11 units higher during the day than at night in the kelp canopy (Fig. 2a, Table 1), and we observed the largest diel fluctuation in pH in the kelp canopy (0.85 pH units) compared to the other habitats (KB: 0.48, US: 0.30, UB: 0.43). Kelp canopy pH was on average 0.16 units higher than the kelp benthos pH (Fig. 2a, Table1). The average diel fluctuations in pH within the urchin barrens did not differ between the surface and the benthos (Fig. 2b, Table 1). When comparing across habitats, pH was 0.11 units higher in the kelp canopy than the urchin barren surface during the day (Fig. 2c, Table 1), but was not different at night (Fig. 2c, Table 1).

**Table 1:** Day and night comparisons of both pH and dissolved oxygen (DO) among kelp canopy (KC), kelp benthos (KB), urchin barren surface (US) and urchin barren benthos (UB).

Comparison	pH		DO	
	*t*	*p*	*t*	*P*
KC_day_ vs KC_night_	**4.69**	**< 0.001**	**7.57**	**< 0.001**
KB_day_ vs KB_night_	0.591	0.555	0.404	0.222
US_day_ vs US_night_	0.079	0.937	0.261	0.794
UB_day_ vs UB_night_	0.118	0.906	0.248	0.206
KC_day_ vs US_day_	**4.53**	**< 0.001**	**7.35**	**< 0.001**
KC_night_ vs US_night_	0.258	0.797	0.448	0.655
KC_day_ vs KB_day_	**7.1**	**< 0.001**	**13.41**	**< 0.001**
KC_night_ vs KB_night_	**3.15**	**0.002**	**6.25**	**< 0.001**
KB_day_ vs UB_day_	0.118	0.906	1.07	0.286

Bolded values denote statistical significance.

**Figure 3:**
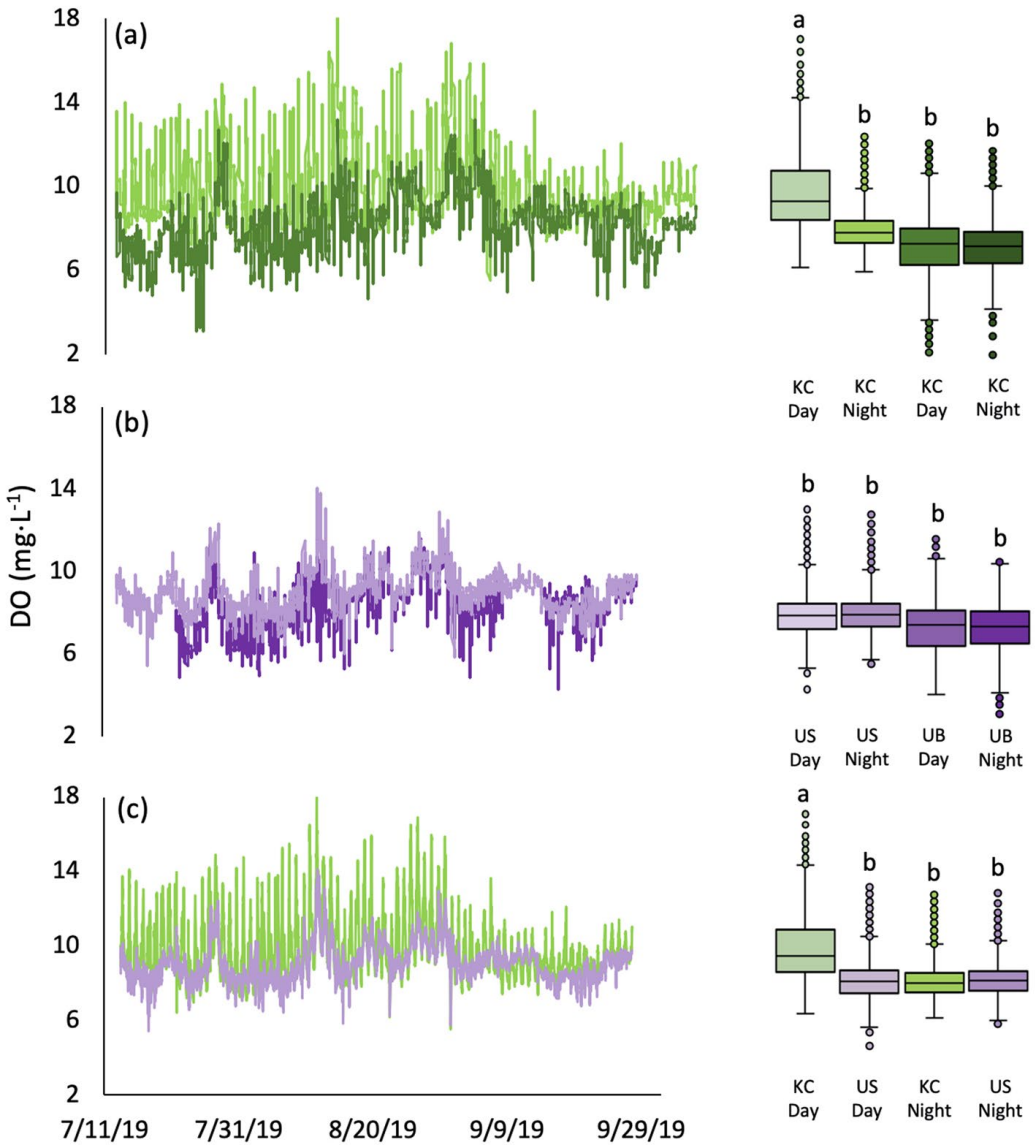
Spatiotemporal variation in dissolved oxygen (DO) in (**a**) kelp canopy (KC-light green) vs. kelp benthos (KB-dark green); (**b**) urchin barren surface (US-light purple) vs. urchin barren benthos (UB-dark purple); (**c**) kelp canopy (KC-light green) vs. urchin barren surface (US-light purple). Each panel includes fluctuations in DO through time (left), and average habitat-specific day and night DO’s (right). Letters denote significant pairwise differences (*P* < 0.001).

DO was on average 16% higher during the day than at night in the kelp canopy (Fig. 3a, Table 1) and exhibited the largest diel fluctuation (11.91 mg L^−1^) relative to other habitats (KB: 7.43, US: 2.94, UB: 3.10). DO did not vary between day and night in the kelp benthos, but kelp canopy DO was 23% higher than the kelp benthos (Fig. 3a, Table 1). DO was not different between the urchin barren surface and urchin barren benthos, regardless of diel period (Fig. 3b, Table 1). When comparing across habitats, DO was 16% higher in the kelp canopy during the day than the urchin barren surface during the day, but not different from those habitats at night (Fig. 3c; Table 1). Seawater temperature was positively correlated with both pH and DO, but the strength of those relationships varied among habitats (see Supplementary Fig. S2 online).

### Variation in seawater chemistry between the kelp interior and kelp edge

**Figure 4:**
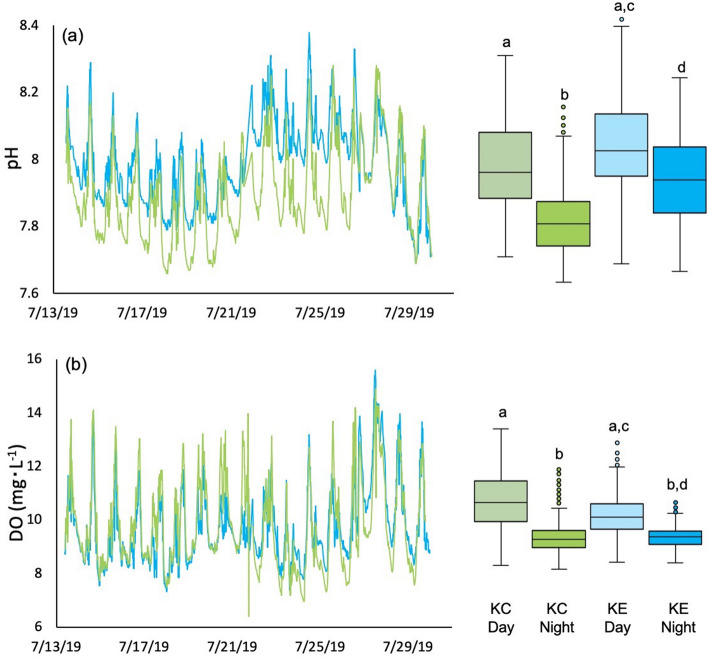
Spatiotemporal variation in (**a**) pH and (**b**) dissolved oxygen (DO) in kelp canopy (interior) (KC-light green) and kelp edge (KE-light blue); boxplots are average habitat-specific day and night parameters. Letters denote significant pairwise differences (*P* < 0.001).

Although we observed the largest diel fluctuations in pH in the kelp canopy, the kelp edge (KE) on average had a higher pH during the day compared to the canopy interior, but only by 0.05 units (Fig. 4a, Table 2). pH at the kelp edge reached as high as 8.38 during the day, and as low as 7.69 at night (Fig. 4a, Table 2). The biggest difference between the kelp canopy interior and the kelp edge came at night, where the kelp edge had a pH 0.11 units higher than that of the kelp canopy (Fig. 4a, Table 2). DO ranged from 7.32 to 15.60 mg L^−1^ at the kelp edge (Fig. 4b, Table 2). Although DO was higher by 0.8 mg L^−1^ in the kelp canopy compared to the kelp edge during the day, DO was similar at the night in both habitats (Fig. 4b, Table 2).

**Table 2:** Day and night comparisons of both pH and dissolved oxygen between kelp canopy interior (KC) and kelp edge (KE).

Comparison	pH		DO	
	*t*	*P*	*t*	*p*
KC_day_ vs KC_night_	**3.79**	**< 0.001**	**4.72**	**< 0.001**
KE_day_ v KE_night_	**2.34**	**0.026**	**2.30**	**0.029**
KC_day_ v KE_day_	1.27	0.213	1.49	0.148
KC_night_ v KE_night_	**2.91**	**0.007**	0.09	0.930

Bolded values denote statistical significance.

During comparable periods (13–29 July 2019), temperature explained only 10% of the variation in pH in the kelp edge, and 18% of the variation in pH in the kelp canopy interior. Temperature explained a similar amount of variation in DO at both the kelp edge (16%) and in the canopy interior (15%).

## Discussion

Our data indicate that small, fragmented kelp patches are capable of altering local seawater chemistry, but only at the kelp canopy. Observed daily fluctuations in pH in and out of kelp patches were consistent with diel patterns of photosynthesis and respiration by bull kelp and differences in pH, pCO_2_, and Ω aragonite were similar in magnitude to those observed in large, intact kelp forests (Table 3). Thus, kelp forest habitats, regardless of size, are capable of modifying local seawater chemistry, which may have implications for calcifying organisms. Such effects are sustained by the physical density of kelp (e.g., frond or stipe density), which inhibits water flow and mixing^35–37^ and so kelp density may be a better predictor of seawater chemistry dynamics in a kelp forest than the areal coverage of the forest^38^.

**Table 3:** Comparison of seawater carbonate chemistry parameters between historically large and persistent kelp forests in the northeastern Pacific and kelp forest fragments at Portuguese Beach and Trinidad Harbor in northern California.

Authors:	Koweek et al. 2017		Murie and Bourdeau			Pfister et al. 2019		Murie and Bourdeau		
	Difference					Difference				
Variable	Comparison	Lover’s Point-Julia Platt	Portuguese	Trinidad	Average (PB and TH)	Comparison	Strait of Juan de Fuca	Portuguese	Trinidad	Average (PB and TH)
DIC	Canopy–Benthos	− 70.00	− 3.61	− 72.61	− 38.11	Inside–outside	NA	− 7.03	116.19	54.58
TA	Canopy–Benthos	− 6.00	45.78	− 38.77	3.51	Inside–outside	6.39	30.88	147.23	89.06
pH	Canopy–Benthos	0.12	0.11	0.07	0.09	Inside–outside	0.09	0.08	0.08	0.08
p Co_2_	Canopy–Benthos	− 144.00	− 130.87	− 80.85	− 105.86	Inside–outside	− 167.10	− 84.54	− 51.49	− 68.02
Ω Ar	Canopy–Benthos	0.61	0.44	0.28	0.36	Inside–outside	0.20	0.35	0.34	0.34
Ω Ca	Canopy–Benthos	NA	0.69	0.44	0.56	Inside–outside	0.31	0.54	0.53	0.53

There were several similarities between our small kelp fragments and large forests. pH, Ω aragonite, and Ω calcite all increased, and *p*CO_2_ decreased, in the kelp canopy during the day relative to other habitats. The average daily pH differences between the surface and benthos of kelp fragments that we observed in our study (PB = 0.11, TH = 0.09) were similar to those documented in central California’s kelp forest (0.12;^15^), and the pH differences in and outside of kelp fragments (PB and TH = 0.09) were similar to that in Washington’s large kelp forests (0.08 pH units). Similarly, Ω aragonite was higher and *p*CO_2_ lower inside than outside of kelp fragments (0.5 units and 131 µatm) to a similar degree to the large Washington kelp forests (0.2 units and 144 µatm;^16^). In addition, although average daily differences in carbonate chemistry in and out of kelp fragments in our study were similar to differences in and out of large kelp forests, overall variation in pH was generally greater (kelp canopy 7.47–8.32) in our kelp fragments, than those recorded in large, intact kelp forests (7.7–8.33^15^; 7.88–8.12^39^, 7.78–8.12^40^, 7.7–8.25^41^, 7.65–8.39^14^; and 7.53–8.19^16^).

Whereas we found evidence for photosynthetic modification of seawater chemistry in the kelp canopy, we did not detect evidence of calcification from our TA data, in the kelp forest or elsewhere. Calcification increases *p*CO_2_ by depleting bioavailable CO_3_ and therefore reducing TA^42^. We therefore expected that TA would have been lower in the benthos relative to the surface in both the kelp forest and the urchin barren due to calcification. The lack of differences in TA could indicate that the chemical signature of calcification was small in the kelp (and urchin) benthos. However in coastal areas organic alkalinity can contribute to TA and without direct measurements of alkalinity in the environment, it is difficult to infer a calcification effect^43^.

Although not significant, there was a slight trend for lower DIC in the kelp canopy compared to the kelp benthos, which is consistent with the decrease in *p*CO_2_ in the kelp canopy. The relatively weaker decrease in DIC in the canopy compared to *p*CO_2_ could be because DIC comprises CO_2_, HCO^3−^, and CO_3_^2−^, and so if CO_3_^2−^ is added to the system in the benthos (e.g., due to the dissolution of calcifying invertebrates or algae in highly acidic benthic waters) then we would end up with a higher DIC than expected in the benthos, negating significant surface-benthos differences. However, this was not the case. Instead, for a given amount of *p*CO_2_, there was less DIC in benthic compared to surface habitats (ANCOVA Habitat*DIC: *F*_1,3_ = 2.98, *P* = 0.035). Interestingly, this pattern (in contrast to the TA data) does suggest a reduction in CO_3_^2−^ in the benthos due to calcification by benthic organisms.

Although chl *a* concentration, which we used as a proxy for phytoplankton abundance, was higher in the kelp fragments than in the adjacent urchin barrens at both PB and TH in 2018, we observed no difference in chl *a* concentration between the kelp forest and urchin barren in PB in 2019. Regardless, we suggest that any effect of phytoplankton-driven modification of seawater chemistry should be small compared to the effects of kelp. For example, Pfister et al.^16^ estimated that for a 7 m^3^ volume of water, carbon fixation by phytoplankton outside of kelp beds (where it is not shaded by kelp or limited by nutrient competition with kelp) is small (0.56 g) compared to carbon fixed by canopy kelp (2.0–9.0 g)^44^. It follows then that photosynthetic modification of seawater by phytoplankton in the canopy, where kelp reduce nutrient concentrations and shade the water column^32,34^, would be relatively low compared to the effects of kelp. We do note that recent studies have shown that carbon fixing microbes can also be abundant in the kelp canopies^16,45^ and that productivity estimates based on chl *a* pigment concentrations or kelp biomass will be an underestimate. If kelp fragment canopies also increase microbial diversity relative to surrounding habitats, then they may also increase metabolic opportunities, including carbon fixation by microbes^16,46^. Regardless of whether kelp per se is the sole driver of seawater chemistry variation or microbes and phytoplankton contribute to the canopy effect, fragmented kelp patches would seem capable of both directly and indirectly modifying their local chemical environment.

Non-biological processes (e.g., upwelling, water flow, etc.) can also contribute to variation in local seawater chemistry in and around kelp forests^47,48^. For example, during periods of upwelling along the coast of California, kelp forests and adjacent coastal habitats are exposed to incursions of cold, nutrient rich, acidic and oxygen-poor water^49,50^. pH, DO, and temperature are highly correlated in upwelled coastal water^49,50^ and so if upwelling was driving the observed variation in seawater chemistry, we would expect to see these strong relationships between temperature and pH and DO maintained across habitats. However, examination of the relationships between temperature and both pH and DO, revealed that temperature explained less variation in pH and DO in the kelp canopy than in other habitats. For example, seawater temperature explained 18% more of the variation in pH and 29% more of the variation in DO in the kelp benthos compared to the kelp canopy. Further, seawater temperature explained 44% more variation in pH in the urchin barren benthos compared to the kelp canopy. Taken together, these data suggest that the metabolic activity of the kelp play a more important role in driving local seawater dynamics than upwelling-driven temperature.

Another physical process that can play a large role in altering local seawater chemistry is water flow and mixing^51^, which depend on exposure to waves and currents, temperature, and depth^15^. We controlled for depth effects by placing each sensor mooring at the same depth in both kelp and urchin barren habitats. We also attempted to keep our sampling locations as similar as possible in terms of wave and current exposure by choosing kelp forest fragments and adjacent urchin barrens that were in close proximity to one another and protected from prevailing northwest swells by prominent headlands. Clod cards fastened to our sensor moorings lost an average of 75% of their mass in the kelp interior, 76% in the kelp edge, and 77% in the urchin barren, after 48 h. Although plaster dissolution does not provide an actual measurement flow speed in each habitat, they have been shown to be a reliable proxy for mass transfer, which is affected in part by flow regime^52^. It is therefore possible to assume that the main residual water flow was similar among habitats. However, local hydrodynamics could be very different in, out, and at the edge of kelp patches because blade density influences the water flow under the canopy^53^. Because kelp can attenuate flow both along and across its boundaries^35–37^, we suggest that any local effects the kelp has on the local seawater flow and chemistry^53^ are likely to persist—even in a fragment.

As with previous studies of large kelp forests^14–16^, canopy-benthos differences in small kelp patches were evident from our data, and they are suggestive that photosynthesis/respiration played an important role in driving the seawater chemistry variability in the kelp canopy. However, unlike previous observations of kelp forests where *Macrocystis pyrifera* dominates^15^, we did not see evidence of a strong photosynthetic effect in the understory algae, relative to adjacent urchin barren benthos, despite its strong presence (mean understory canopy (mainly *Desmarestia* and *Pterygophora*); percent cover 86 ± 10 sd). We provide four alternative hypotheses as to why. First, the resolution of our sampling (e.g., 1 m above the benthos) might have prevented us from being able to quantify an understory effect (e.g.,^15^). Second, *Nereocystis* is very effective at creating a canopy habitat that inhibits high light intensity from reaching the bottom during the day; indeed, we found that the kelp forest benthos received 11% less light intensity (lum ft^−2^) than the kelp canopy. Although understory kelps are typically low-light acclimated [e.g.,^54^] and should be able to photosynthesize even if light is attenuated by the canopy, it may be that the rate of photosynthesis was not great enough to alter local seawater chemistry. Thirdly, cold, acidic upwelled water did not mix vertically in our habitats and thus similar low pH’s at the kelp forest and urchin barren benthos are due to benthic incursion of acidic upwelled seawater^49,50^. Finally, similarly-low pHs at the kelp and urchin barren benthos may be due to the presence of *Desmarestia* spp. (acid weed) in the understory of our kelp patches. *Desmarestia * is quite abundant in our kelp patches, and when disturbed, will release sulfuric acid, which can lower the pH of surrounding seawater^55^. Further studies are needed to distinguish among these alternative hypotheses.

As climate change-associated stressors continue to impinge on coastal habitats, kelp forests in northern California may continue to decline^56–58^, increasing the abundance of fragmented patches relative to intact forest habitat. Increases in forest fragmentation leads to an increase in the ratio of perimeter to interior (i.e., edge) habitat, which can have implications for forest carbon dynamics^29–31^. In the summer, when we sampled and when kelp canopies were well-developed, fronds at the forest’s edge had larger, healthier-looking blades than those in the forest’s interior, suggesting higher carbon fixation and growth rates in edge fronds. Light availability in the kelp forest’s interior may also be low enough that nutrient uptake and photosynthesis is limited. Consistent with these observations, we found that during the day, seawater at the kelp edge was on average 0.05 pH units higher than seawater in the kelp interior. However, the biggest difference in pH between the kelp edge and interior was at night. On average pH was 0.11 units higher at the kelp edge at night than the interior. We hypothesize that higher pH at the kelp edge is due to longer exposure to light at the kelp edge compared to the interior. Our data supported this in that light intensity during daylight hours was > 350 lum ft^−2^ 65% of the time at the kelp edge compared to 25% in the kelp interior.

Understanding the role of foundation species like kelp in creating OA refugia through metabolic activity is increasingly important to understand as OA and its effects on marine organisms are predicted to worsen^59^. Organismal growth, metabolism, and behavior have all been shown to be negatively affected in seawater with pH lower than 7.75. For example, sea urchin larvae are 7–13% smaller when exposed to pH below 7.7^60^, and juvenile rockfish are 17% slower when pH drops to 7.5^61^. Our data indicate that seawater in the kelp canopy had a pH of 7.75 or greater 75% the time, whereas seawater pH in the kelp benthos and adjacent urchin barren surface was greater than 7.75 only 59 and 52% of the time, respectively. Further, organisms may experience sublethal effects of low oxygen under current conditions. For example, sea urchins exhibit 39–47% declines in grazing at 5.5 mg l^−1^ DO^62^. We found that the kelp benthos and urchin barren benthos reached these levels of moderate hypoxia only 3% and 0.8% of the time, respectively, whereas the kelp canopy never reached these levels.

Based on our findings, we hypothesize that the combination of physical and biological processes in kelp fragments may create natural diurnal and seasonal refugia for canopy-dwelling organisms^63,64^. However, because we sampled in the summer, when bull kelp would have the biggest effect on seawater chemistry^65^, the magnitude we observed is likely temporary and only during daytime during the spring and summer^17,18^. Further, although animals may take advantage of periods of pH refugia from the kelp during the day, large fluctuations during day-night cycles in the kelp^18,66^ could create a stressful environment, as in coral reef communities^67^. Alternatively, fluctuations could mitigate OA impacts as for calcifiers in algal boundary layers^53,63,68^. If calcifying organisms can calibrate their calcification to periods of high pH^9^, these diel and seasonal cycles could be critical for organismal fitness and performance. Consequently, we need to understand kelp’s ability to modulate local environments across multiple spatiotemporal scales if we are to effectively manage critical coastal ecosystems in the face of OA, climate change, and kelp deforestation.

## Methods

### Site selection and characterization

**Figure 5:**
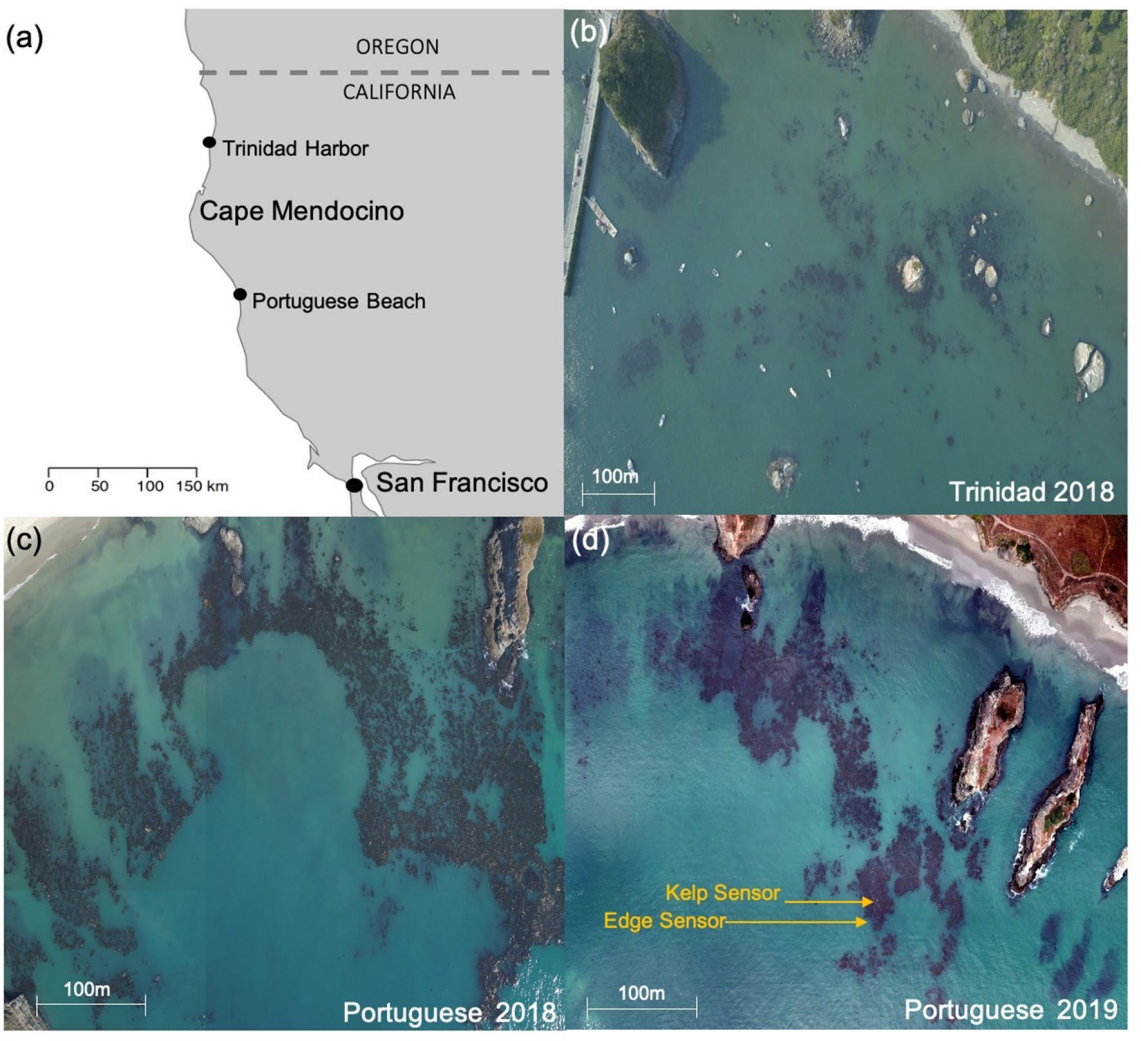
Study locations of two fragmented kelp forests in (**a**) northern California (map generated in R [v3.6.1]), and kelp canopy coverage in (**b**) Trinidad Harbor in 2018 and Portuguese Beach kelp canopy in (**c**) 2018 and (**d**) 2019. The main images for (**b**–**d**) are an orthomosaic map comprised of several aerial photographs captured by an unmanned drone (by Jeremy McFarland) and merged and geometrically corrected such that the scale is uniformly adjusted for topographic relief, lens distortion, and camera tilt. Kelp interior and edge sensor arrays indicated with arrows in (**d**).

Our study sites were Trinidad Harbor (TH) in Trinidad, CA (41.0ʹ N, 124.1ʹ W) and Portuguese Beach (PB) in Mendocino County, CA (39.2ʹ N, 123.5ʹ W) (Fig. 5a). Sites were selected for the presence of bull kelp and adjacent urchin barrens. TH is protected from prevailing northwest swells by Trinidad Head, a large rocky promontory. Bull kelp is aggregated on large boulders, creating small kelp patches (Fig. 5b) with the urchin barren 1,568 m to the south. The kelp at PB is mainly on a flat rocky reef divided by wide sand channels, with more evenly-spaced stipes (Fig. 5c). As with TH, kelp at PB is protected from northwest swells by the Mendocino Headlands; the urchin barren is ~ 100 m from the kelp.

To characterize habitats at each site, we used aerial and SCUBA surveys at TH and PB in 2018, and PB in 2019 (Fig. 5d), due to the lack of kelp at TH in 2019. We estimated kelp canopy area from digital images captured from an unmanned drone (DJI Mavic Pro) and analyzed with ArcGIS Pro (v2.6). Radiometric orthomosaic techniques and spatial classifications allowed us to minimize water reflection and isolate bull kelp canopy in the images from areas without kelp. To estimate kelp and urchin densities, we did a series of transect surveys, placing the first 30 m × 2 m transect 1 m inside the edge of the kelp at a depth between 6 and 7 m, depending on the tide, and placing subsequent transects 5 m apart and parallel to the first; ranging from 4 to 5.5 m depth. Using this depth contour, we placed three transects in the adjacent urchin barren. Divers counted algal stipes > 30 cm and urchins > 2.5 cm along transects using PISCO protocols (https://www.piscoweb.org/).

### Habitat variation in seawater chemistry

To measure spatial variation in carbonate chemistry at the surface and the benthos in and out of kelp, we collected in situ water samples at PB (n = 127) and TH (n = 222) in 2018. Samples were taken weekly in TH and biweekly at PB June–October. We collected samples 1 m below the surface (m.b.s) (where most kelp biomass resided) and 1 m above the bottom (m.a.b) to quantify bull kelp’s capacity to alter seawater chemistry in the canopy vs. benthos. This was repeated in urchin barrens, creating four distinct habitats: kelp canopy (KC), kelp benthos (KB), urchin barren surface (US), and urchin barren benthos (UB). Samples were collected by hand in 350 mL amber glass bottles to inhibit light entry, and then, as quickly as possible on shore, poisoned with 100 µL of saturated mercuric chloride (HgCl_2_) to halt biological activity. Four replicate samples were always collected from each habitat between 1000 and 1400 h and within 60 m from one another; the order of habitats was randomized. *p*CO_2_ and DIC measurements were made on a CO_2_ analyzer (“Burkeolator”) via gas equilibration and stripping, respectively, followed by infrared detection^69^), and modified for discrete samples^70^. To ensure accuracy, gas and liquid standards included the complete range of values for ocean seawater: primary gas standards were precise to ± 1% with nominal *p*CO_2_ of 200, 800, and 1,500 ppm; liquid standards were prepared to a precision of 1.0 × 10^–6^ with nominal DIC of 1,800, 2,200, and 2,500 mol kg^−1^. Samples were run only when both gas and liquid standard calibration curves were highly linear (*R*^2^ ≥ 0.999). Calibration curves for both DIC and *p*CO^2^ were run before and after each batch of samples; values calculated using before- and after-calibration curves never differed > 0.2%. To calculate complete carbonate chemistry (pH [total scale], total alkalinity [TA], Ω aragonite, and Ω calcite) we used ‘seacarb’^71^ in R (v3.2.10)^72^. We did not measure silicate and phosphate concentrations but tested whether including the most extreme values recorded in our region^73^ affected our estimates of pH, TA, Ω aragonite, and Ω calcite. Including silicate and phosphate concentrations in the ‘seacarb’ calculations did not affect our estimates of pH, Ω aragonite, and Ω calcite, and only slightly changed our estimates of TA (− 4.27e−6). Therefore, we set silicate and phosphate concentrations to zero.

### Temporal variation in seawater chemistry

To quantify temporal variation in surface and benthic seawater chemistry in and out of kelp and to confirm that diel fluctuations in pH and dissolved oxygen (DO) were consistent with photosynthetic activity by bull kelp, we deployed sensor arrays 1 m.b.s and 1 m.a.b in each of the four habitats at PB from June to September 2019 (a schematic of sensor arrays and placement is in Supplementary Fig. S1 online). Each sensor array consisted of a HOBO pH (MX2501), DO (U26-001), and conductivity (U24-002-C) logger (Onset, USA). Sensors were randomly rotated among habitats throughout the study to remove individual sensor effects. Sensors logged data continuously at 15 min intervals throughout the study. We calibrated sensors weekly. pH sensors were calibrated using a 3-point calibration and presented on the total hydrogen ion concentration scale after cross-calibration with Tris buffers^31^. Calibration samples produced an accuracy of 0.03–0.08 units on average for each logger. We also collected weekly discrete water samples directly adjacent to sensors, allowing us to verify sensor readings. DO sensors were calibrated using a one-point calibration at 100% saturation, corrected for salinity over time (HOBOwarePro v3.7.16) and found accurate up to ± 0.5 mg L^−1^.

### Variation in seawater chemistry between the kelp interior and edge

To quantify diel fluctuations in pH and DO in the interior and edge of the kelp canopy, we also deployed sensor arrays 1 m.b.s and 1 m.a.b at the kelp edge at PB in summer 2019. Due to the expansion of the kelp forest perimeter at the end of July, and the senescence of interior kelp in at the end of August, we only used data from July 13 to 29 to ensure we were only comparing differences between the kelp edge and the healthy interior, so caution should be taken in extrapolating these results.

### Among-habitat variation in chlorophyll, flow, and light

We collected weekly seawater samples in 250 mL brown plastic bottles from the kelp canopy interior and the urchin barren surface (n = 5 week^−1^ habitat^−1^). Samples were immediately brought back to the lab for vacuum filtration (< 10 psi or 500 mm Hg) through 47 mm GF/F filters for chl *a* analysis. Filters were placed in 15 mL borosilicate tubes with 4 ml of 90% acetone and stored in a spark-free freezer for 18–24 h prior to analyses on a fluorometer with acidified chlorophyll module and solid standard (Turner Designs Trilogy). Fluorometric measurements, done after equilibrating samples to room temperature (~ 30 min) and before and after the addition of 10% HCl, were compared to a solid standard to calculate chl *a*.

We used the dissolution of clod cards (plaster blocks) as an integrated measure of water movement in each habitat^52^. Calibrations were carried out in flow-through seawater in the lab, but because plaster dissolution rate depends on temperature, turbulence, and flow type (fluctuating, steady, mixed^74^; it can only provide approximate measures of flow speed. However, plaster dissolution is a good indicator of mass transfer, which depends on flow speed, type, and turbulence ^74^. Blocks were fastened to sensor arrays in each habitat (KC interior, KE, and UB) at three depths (1 m.b.s, 1 m.a.b., and midpoint of the water column). After ~ 48 h, blocks were collected, dried at 65 °C, and weights recorded. Dissolution was quantified as proportion of initial mass lost.

Two light loggers (HOBO Pendant Temp/Light MX2202) were fixed horizontally with the light sensor facing upward to the top of each sensor array in each habitat. Although sensors recorded light intensity in units of Lux, we were only interested in relative differences in light intensity among habitats and so we did not calibrate these data to PAR.

### Statistical analyses

To assess spatial variation in a pH, *p*CO_2_, Ω aragonite, Ω calcite, DIC, and TA, we took the average of the measured (*p*CO_2_ and DIC) or calculated (all other parameters) values from each of the four replicate samples from each habitat on a given day, and calculated the difference in these values between all habitats (e.g., kelp canopy minus kelp benthos, urchin barren surface minus urchin barren benthos, etc.). We constructed 95% confidence intervals around the average daily differences between habitats to assess statistical significance. 95% CI are more informative than traditional significance tests and allow hypothesis testing while providing a range of values that reflect the uncertainty in the difference estimate^75,76^. If 95% CI did not overlap zero, the average daily difference for a given parameter was significant at α = 0.05.

Temporal pH and DO data from each habitat were converted to daily day (0800–2000 h) and night (2001–0759 h) means and differences between means were analyzed with paired t-tests. We assessed relationships between seawater temperature and pH and DO in each habitat using least squares regression. We used paired t-tests to analyze between-habitat differences in chl *a*, and ANOVA to analyze among habitat differences in water motion. Bonferroni corrections were applied to *p*-values for repeated t-tests. Analyses were done in R (v3.6.1) ^72^.

## Supplementary information

Supplementary file1 (DOCX 8334 kb)
