# Supplementary material for: Fragmented kelp forest canopies retain their ability to alter local seawater chemistry
Source: Sci Rep. 2020 Jul 20;10:11939. doi: 10.1038/s41598-020-68841-2 (PMC7371639; doi:10.1038/s41598-020-68841-2)
Supplement: Supplementary file 1 — Supplementary file1 (DOCX 8334 kb) [file 41598_2020_68841_MOESM1_ESM.docx]

**Supplementary Information**

**Title:** Fragmented kelp forest canopies retain their ability to alter local seawater chemistry

**Authors:** Kindall A. Murie^1,2*^ & Paul E. Bourdeau^1,2^

**Author affiliations:** ^1^Telonicher Marine Laboratory, Humboldt State University, Trinidad, USA.

^2^Department of Biological Sciences, Humboldt State University, Arcata, USA.

***email:** [Kindall.Murie@humboldt.edu](mailto:Kindall.Murie@humboldt.edu)

**
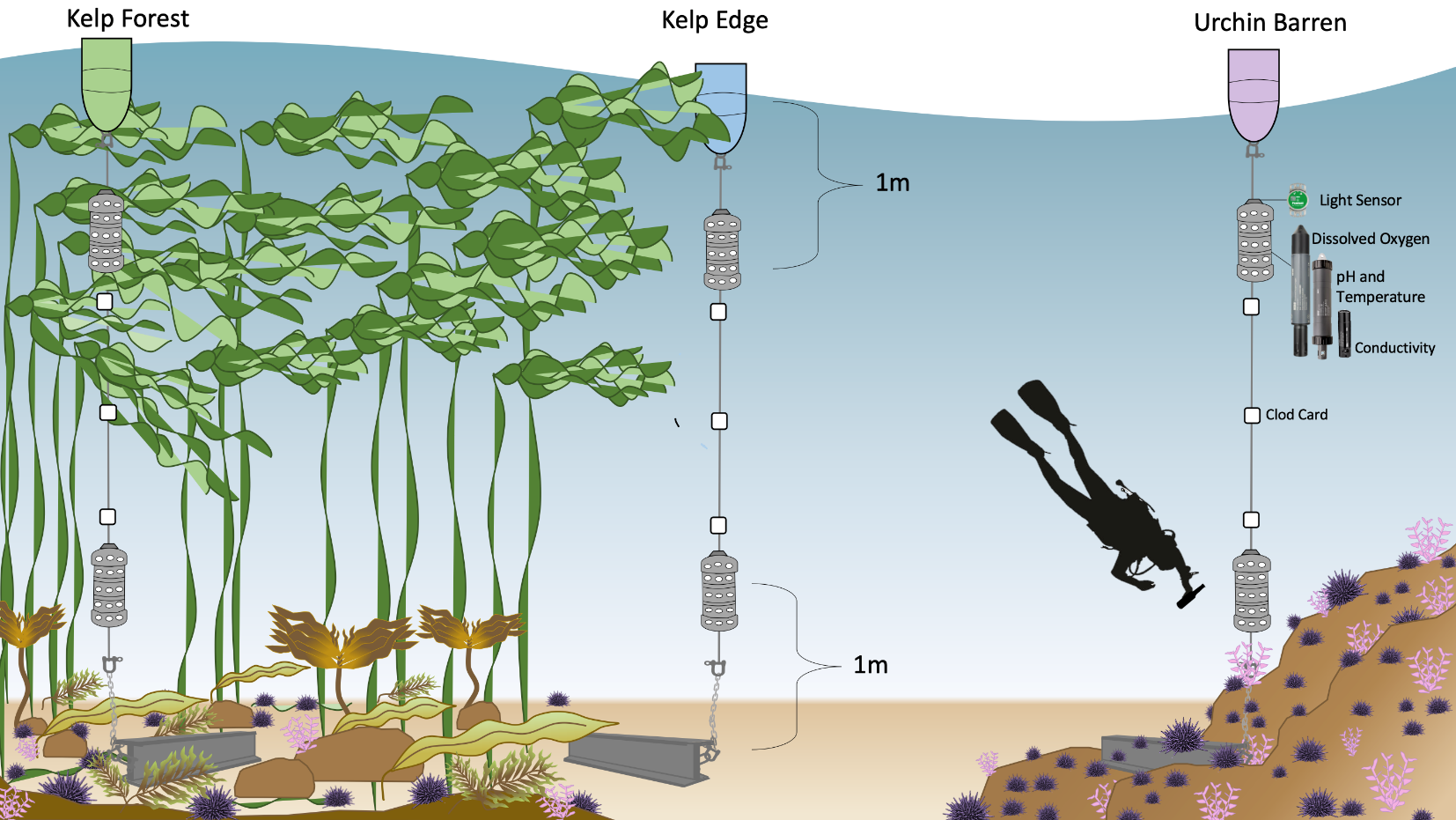
Supplementary Figure S1**

Supplementary Figure S1

Schematic of sensor array deployment at Portuguese Beach from July to September 2019. Sensor arrays were located 1 meter below the surface (m.b.s.) and 1 meter above the bottom (m.a.b) in three different habitats: interior of kelp forest, edge of kelp forest, and adjacent urchin barren. Anchored with an I-beam and subsurface buoy, each sensor array was attached to the steal cable with a protective PVC housing with large perforations to allow flow. Each sensor array contained a pH, DO, conductivity, and light logger (HOBO®, Onset®, USA). Light loggers were mounted horizontally to the Square PVC cap of the sensor arary housing to orient the sensor toward the surface. DO, pH, and conductivity loggers were oriented vertically within the housing with sensors facing toward the benthos. All sensors arrays were placed at the same depth in all three locations, with max depth of 6m at high tide.

**Supplementary Figure S2**

Supplementary Figure S2

(a) Spatiotemporal variation in temperature in kelp canopy (KC-light green), kelp benthos (KB-dark green), urchin barren surface (US-light purple), and urchin barren benthos (UB-dark purple). Relationship between temperature and (b) pH and (c) DO in the kelp canopy ( ), kelp benthos ( ), urchin barren surface ( ), urchin barren benthos ( ). *R*^2^’s from OLS regression for temperature-pH relationship: KC = 0.03; KB = 0.21, US = 0.23, UB = 0.47; temperature-DO relationship: KC = 0.05, KB = 0.34, US = 0.17, and UB = 0.31. All *P*’s < 0.001.
